# Tumour deposit count is an independent prognostic factor in colorectal cancer—a population-based cohort study

**DOI:** 10.1093/bjs/znae309

**Published:** 2024-12-30

**Authors:** Simon Lundström, Erik Agger, Marie-Louise Lydrup, Fredrik Jörgren, Pamela Buchwald

**Affiliations:** Department of Surgery, Skåne University Hospital, Malmö, Sweden; Department of Clinical Sciences, Lund University, Lund, Sweden; Department of Surgery, Skåne University Hospital, Malmö, Sweden; Department of Clinical Sciences, Lund University, Lund, Sweden; Department of Surgery, Skåne University Hospital, Malmö, Sweden; Department of Clinical Sciences, Lund University, Lund, Sweden; Department of Clinical Sciences, Lund University, Lund, Sweden; Department of Surgery, Helsingborg Hospital, Lund University, Helsingborg, Sweden; Department of Surgery, Skåne University Hospital, Malmö, Sweden; Department of Clinical Sciences, Lund University, Lund, Sweden

## Abstract

**Background:**

Tumour deposits are a prognostic factor for overall survival and distant metastasis in lymph node-negative colorectal cancer. However, the current TNM staging system does not account for the presence of tumour deposits in lymph node-positive colorectal cancer, or for the presence of multiple deposits. This study aimed to investigate the prognostic effect of tumour deposit count in patients with colorectal cancer.

**Methods:**

Patients who underwent curative surgery for colorectal cancer between 2016 and 2019 were identified nationwide from the Swedish Colorectal Cancer Registry. Patients with undisclosed tumour deposit status/count and stage IV disease were excluded. Univariable and multivariable Cox regression analyses were used to assess the prognostic effect of tumour deposit count on overall survival and distant metastasis adjusted for age, sex, neoadjuvant treatment, and number of positive lymph nodes.

**Results:**

Of 18 913 patients assessed, 14 154 patients were analysed with tumour deposits (TDs) present in 1702 (12%) patients. Patients were stratified by tumour deposit count (0, 1, 2, 3, 4, and ≥5 TDs). Increased tumour deposit count was associated with decreased 5-year overall survival (79%, 70%, 61%, 66%, 50%, 49%) and increased 5-year risk for distant metastasis (14%, 26%, 35%, 41%, 48%, 54%) respectively. Tumour deposit count remained an independent negative prognostic factor after multivariable Cox regression analysis.

**Conclusion:**

Tumour deposit count is a negative prognostic predictor of both overall survival and distant metastasis in colorectal cancer, independent of positive lymph nodes or neoadjuvant treatment. These findings suggest that tumour deposit count should be integrated into the TNM staging regardless of lymph nodes status to improve prognostic accuracy.

## Teaser

This study investigates the prognostic significance of multiple tumour deposits (TDs) in CRC using population-based data from the Swedish Colorectal Cancer Registry. Our findings demonstrate that TD count is a prognostic marker for overall survival and distant metastasis, independent of neoadjuvant treatment and number of positive lymph nodes.

## Introduction

Colorectal cancer (CRC) is the third most common malignancy worldwide and is staged according to the TNM staging system, which is considered the single most important prognostic factor for overall survival, distant metastasis, and local recurrence^1,2^. Despite recent diagnostic and therapeutic advances, the 5-year mortality for CRC remains at 65%, and more accurate prognostic and therapeutic tools are called for^3,4^.

Tumour deposits are discrete, discontinuous tumour nodules within the colorectal mesentery without any identifiable lymphatic, vascular, or neural structures^2,5^. Tumour deposits are observed in roughly 15–25% of CRC, and multiple studies have shown a strong correlation with impaired survival and increased risk of distant metastasis in colon and rectal cancer cohorts^5–11^. Tumour deposits often exist synchronously with positive lymph nodes and other negative prognostic histopathological findings such as high tumour grade, perineural growth, and extramural vascular invasion (EMVI)^12,13^

With the introduction of the N1c-stage in the seventh TNM edition, the presence of tumour deposits was assigned prognostic value in lymph node-negative CRC^2^. However, the current TNM staging system does not account for the presence of tumour deposits in lymph node-positive CRC, or the presence of multiple deposits^2^. Consequently, important clinical information may be overlooked when determining treatment for patients with multiple tumour deposits and/or synchronous positive lymph nodes^14,15^.

The aim of this study was to evaluate if tumour deposit count is a negative prognostic factor for overall survival, distant metastasis, or local recurrence in a national cohort of patients treated for CRC. We hypothesized that an increased tumour deposit count would negatively impact long-term oncological outcomes.

## Methods

### Swedish Colorectal Cancer Registry

The Swedish Colorectal Cancer Registry is a national database that includes 99% of patients with CRC in Sweden and has a high level of data ascertainment^16^. The data set includes over 800 variables with validation of the registry routinely performed^17^. Recurrence data are collected continuously and in conjunction with standardized follow-up at three and five years.

### Study population

**Fig. 1:**
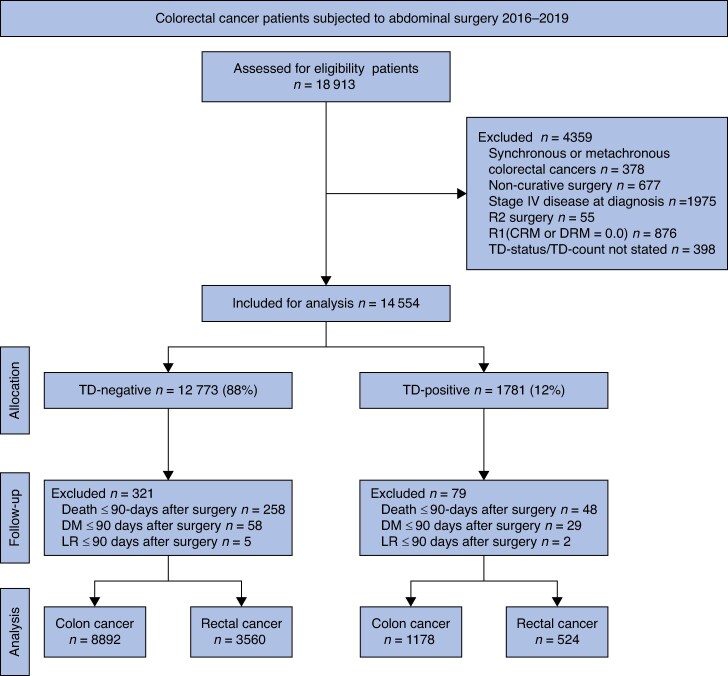
Study flowchart TD, tumour deposit; CRM, circumferential resection margin; DRM, distal resection margin; DM, distant metastasis; LR, local recurrence

Data from the registry were extracted for patients who underwent resectional surgery for a solitary colon or rectal cancer with curative intent between January 2016 and December 2019. Survival data were collected from the Swedish Cause of Death Register on 23 January 2024. A flowchart of the inclusion and exclusion process is presented in *Fig. 1*. Early study outcomes (i.e. mortality or disease progression) within 90 days of surgery were excluded as they were believed to represent cases of postoperative mortality secondary to complications, undiagnosed metastases present at the time of surgery, or palliative surgical resection. After exclusions, patients were stratified by tumour deposit (TD) count (0, 1, 2, 3, 4, and ≥5 TDs).

Tumour deposits were defined according to the seventh or eighth edition of the TNM staging system depending on the year of diagnosis^2,5^. For the seventh TNM edition, tumour deposits were defined as a foci of tumour cells discontinuous from the primary tumour located within the lymphatic drainage of the pericolic or perirectal fat tissue without evidence of lymphatic tissue^5^. In the eighth edition, tumour deposits should, in addition, not contain any neural or vascular tissue^2^. A detailed description of the variables used in the study is presented in Supplementary Method*s*. All histopathological specimens were examined by a gastrointestinal pathologist as part of the clinical routine.

### Primary study outcomes

The primary study outcomes were overall survival, distant metastasis, and local recurrence. Overall survival was defined as the time from primary surgery to death from any cause. Distant metastasis was defined as recurrence of a tumour in the peritoneum or mesenteric lymph nodes remote from the original tumour as documented by clinical, radiological, or histopathological examination. Local recurrence was defined as the recurrence of local extraperitoneal tumour, lymph node tumour growth, intraluminal tumour recurrence, or peritoneal tumour growth below the peritoneal reflection, as documented by clinical, radiological, or histopathological examination. Distant metastasis and local recurrence-free survival was defined as the time from surgery to either the last follow-up, or the occurrence of the outcome event.

### Statistical analysis

Categorical variables were presented as numbers and proportions in percentages. Numerical data were reported as median with interquartile range. Fisher’s exact test with Monte Carlo was used for intergroup comparisons where appropriate.

Kaplan–Meier curves were used to determine the 5-year cumulative overall survival, as well as the 5-year cumulative risk of distant metastasis and local recurrence, with differences investigated using the log-rank test. Uni- and multivariable Cox regression analyses were performed for relative survival, overall survival, distant metastasis, and local recurrence. Confounding variables for multivariable analysis were identified prior to data analysis with the aid of directed acyclic graphs (*Fig. S1*) and limited by the number of outcomes. Adjustments were performed for age, sex, neoadjuvant treatment, and positive lymph nodes. Relative survival was based on overall survival, and additionally adjusted for death of any cause in an age-matched group of citizens using data from the Human Mortality Database (Sweden, life table for men and female by age and year in 1-year intervals).

### Sensitivity and subgroup analysis

Descriptive and Kaplan–Meier survival analyses of the effect of tumour deposit count on the primary outcomes based on tumour location (colon or rectal) were performed. For the combined CRC cohort, Cox regression sensitivity analysis was performed with additional adjustment for clinical stage, pathological stage, tumour grade, perineural growth, vascular/lymphatic invasion, EMVI, and the year of diagnosis. To enable sensitivity analysis, multivariable imputation of the EMVI variable was performed on patients operated in 2016 due to an update of the registry in 2017, where the histopathological variable ‘vascular/lymphatic invasion’ was subdivided into ‘vascular invasion’, ‘lymphatic invasion’, and ‘EMVI’. The imputation was based on sex, age, tumour location, clinical stage, neoadjuvant treatment, year of surgery, pathological stage, number of positive lymph nodes, tumour deposit count, perineural growth, tumour grade, vascular/lymphatic infiltration, vascular invasion, lymphatic invasion, EMVI, local recurrence, time to local recurrence, distant metastasis, time to distant metastasis, length of time in follow-up, and mortality rate. All available data from the data set prior to exclusion were included for the imputation process. Twenty imputations were performed using a seed value of 20 240 525.

All statistical analyses were performed using R version 4.2.1^18^ with RStudio^19^. Kaplan–Meier curves and Cox regressions were created using the *survival* package^20^ and visualizations were generated using the *ggplot2* package^21^. Imputation was performed using the *mice* package^22^ and relative survival was calculated using the *relsurv* package^23^. *P* < 0.05 was considered statistically significant.

### Missing data

Patients with unspecified status due to emigration were censored for relative survival and overall survival in Kaplan–Meier and Cox regression analysis at the day of emigration. Patients with unspecified follow-up status due to missing data were censored for distant metastasis and local recurrence for Kaplan–Meier and Cox regression analysis at day zero.

### Ethical considerations

This study was approved by the Swedish Ethical Review Authority (DNR: 2023-02433-02) and the registry steering committee. Contribution to the registry is voluntary with the possibility to delete personal health data at any time. Registration in the Cause of Death Register is mandatory for all citizens by law and does not require consent.

## Results

### Study population

Between January 2016 and December 2019, 18 913 patients from the registry database fulfilled the inclusion criteria and were evaluated for eligibility (*Fig. 1*). After exclusions, 14 154 (75%) patients remained, among whom 1702 (12%) had evidence of tumour deposits.

Baseline characteristics of tumour deposit-negative and tumour deposit-positive CRC patients are summarized in *Table 1*. Only 495 (29%) tumour deposit-positive patients were classified as N1c. While both TD-positive and TD-negative tumors were more commonly left-sided, the proportion of right-sided tumors was higher among TD-positive cases compared to TD-negative cases. These patients also tended to have higher clinical stage and were more likely to have undergone neoadjuvant therapy.

**Table 1:** Patient and preoperative characteristics for tumour deposit-negative and tumour deposit-positive patients

	Total (*n* = 14 154)	Tumour deposit-negative (*n* = 12 452)	Tumour deposit-positive (*n* = 1702)
**Sex**			
Male	7358 (52.0)	6452 (51.8)	906 (53.2)
**Age**			
Years	73 (65–79)	73 (66–80)	71 (64–78)
**BMI**			
kg/m^2^	26 (23–29)	26 (23–29)	26 (23–29)
**Tumour location**			
Colon	10 070 (71.1)	8892 (71.4)	1178 (69.2)
Left colon	6409/10 070 (63.6)	5763/8892 (64.8)	646/1178 (54.8)
Right colon	3643/10 070 (36.2)	3112/8892 (35.0)	531/1178 (45.1)
Rectum	4084 (28.9)	3560 (28.6)	524 (30.8)
**ASA score**			
ASA 1–2	9013 (63.7)	7877 (63.3)	1136 (66.7)
ASA 3	4438 (31.4)	3948 (31.7)	490 (28.8)
ASA 4	349 (2.5)	308 (2.5)	41 (2.4)
ASA 5	9 (0.1)	8 (0.1)	1 (0.1)
Missing	345 (2.4)	311 (2.5)	34 (2.0)
**Time to surgery**			
Days	35 (24–55)	35 (24–55)	35 (24–55)
**cT stage**			
T1–T2	4140 (29.2)	3872 (31.1)	268 (15.7)
T3	5989 (42.3)	5119 (41.1)	870 (51.1)
T4	1917 (13.5)	1584 (12.7)	333 (19.6)
TX	1871 (13.2)	1671 (13.4)	200 (11.8)
Missing	237 (1.7)	206 (1.7)	31 (1.8)
**cN stage**			
N0	7333 (51.8)	6775 (54.4)	558 (32.8)
N1-2	5780 (40.8)	4794 (38.5)	986 (57.9)
NX	847 (6.0)	717 (5.8)	130 (7.6)
Missing	194 (1.4)	166 (1.3)	28 (1.6)
**cStage**			
Stage I	3317 (23.4)	3129 (25.1)	188 (11.0)
Stage II	3025 (21.4)	2722 (21.9)	303 (17.8)
Stage III	5812 (41.1)	4815 (38.7)	997 (58.6)
Missing	2000 (14.1)	1786 (14.3)	214 (12.6)
**Discussed at a preoperative multidisciplinary conference**			
Yes	12 942 (91.4)	11 440 (91.9)	1502 (88.2)
**Neoadjuvant therapy**			
Yes	2645 (18.7)	2236 (18.0)	409 (24.0)

Continuous values are presented as median (i.q.r.). Categorical values are presented as frequency (%). c = clinical.

Histopathological characteristics of tumour deposit-negative and tumour deposit-positive CRC patients are presented in *Table 2*. Tumour deposit-positive patients more often exhibited synchronous negative prognostic histopathological findings, such as circumferential resection margin involvement, lymphovascular invasion, EMVI, perineural growth, and high tumour grade. Subsequently, tumour deposit-positive patients more often received adjuvant therapy compared to tumour deposit-negative patients (56% *versus* 21% respectively). The median (i.q.r.) follow-up was slightly longer for tumour deposit-negative patients (63 (51–71) months) compared to tumour deposit-positive patients (58 (37–75) months).

**Table 2:** Postoperative histopathology for tumour deposit-negative and tumour deposit-positive patients

	Total (*n* = 14 154)	Tumour deposit-negative (*n* = 12 452)	Tumour deposit-positive (*n* = 1702)
**pT stage**			
pT0	46 (0.3)	45 (0.4)	1 (0.1)
pT1	1155 (8.2)	1138 (9.1)	17 (1.0)
pT2	3034 (21.4)	2933 (23.6)	101 (5.9)
pT3	7706 (54.4)	6674 (53.6)	1032 (60.6)
pT4	2212 (15.6)	1661 (13.3)	551 (32.4)
Missing	1 (0.0)	1 (0.0)	0 (0)
**pN stage**			
N0	8947 (63.2)	8947 (71.9)	0 (0)
N1a	1682 (11.9)	1408 (11.3)	274 (16.1)
N1b	1503 (10.6)	1138 (9.1)	365 (21.4)
N1c	495 (3.5)	0 (0)	495 (29.1)
N2a	865 (6.1)	566 (4.5)	299 (17.6)
N2b	536 (3.8)	277 (2.2)	259 (15.2)
NX	126 (0.9)	116 (0.9)	10 (0.6)
**pStage**			
Stage I	3463 (24.5)	3463 (27.8)	0 (0)
Stage II	5542 (39.2.)	5542 (44.5)	0 (0)
Stage III	5144 (36.3)	3442 (27.6)	1702 (100)
**Positive lymph nodes**			
Number of lymph nodes	0 (0–1)	0 (0–1)	2 (0–4)
**Less than 12 examined lymph nodes**			
Yes	897 (6.3)	801 (6.4)	96 (5.6)
**Tumour deposits**			
None	12 452 (88.0)	12 452 (100)	0 (0)
1	845 (6.0)	0 (0)	845 (49.6)
2	380 (2.7)	0 (0)	380 (22.3)
3	178 (1.3)	0 (0)	178 (10.5)
4	101 (0.7)	0 (0)	101 (5.9)
≥5	198 (1.4)	0 (0)	198 (11.6)
**CRM (mm)**			
0.1–1.0 mm	566 (4.0)	397 (3.2)	169 (9.9)
> 1.0 mm	12 884 (91.0)	11 414 (91.7)	1470 (86.4)
Missing*	704 (5.0)	641 (5.1)	63 (3.7)
**Lymphovascular invasion**			
Yes	4142 (29.3)	3112 (25.0)	1030 (60.5)
**EMVI**			
Yes	1685 (11.9)	1149 (9.2)	536 (31.5)
Missing†	1182 (8.4)	890 (7.1)	292 (17.2)
**Perineural growth**			
Yes	2407 (17.0)	1686 (13.5)	721 (42.4)
**Tumour grade**			
High-grade	2584 (18.3)	2172 (17.4)	412 (24.2)
**Adjuvant therapy**			
Yes	3571 (25.2)	2618 (21.0)	953 (56.0)

Continuous values are presented as median (i.q.r.). Categorical values are presented as frequency (%). c = clinical. p = histopathological. *Negative circumferential resection margin (CRM) but missing distance measurement. †Based on patient from 2016 with positive ‘lymphovascular invasion’ or patients from any year with unknown ‘lymphovascular invasion’ status.

### Overall survival, distant metastasis, and local recurrence

The overall survival, distant metastasis, and local recurrence rates based on tumour deposit count by tumour location (colon or rectum) are presented in *Table 3*. Colon cancer patients generally had lower overall survival compared to rectal cancer patients, although they less frequently experienced distant metastasis or local recurrence. Multiple tumour deposits were consistently associated with a higher risk for mortality, distant metatasis, and local recurrence in both colon and rectal cancers compared to a single tumour deposit (*P* < 0.001).

**Table 3:** The effect of tumour deposit count on overall survival, distant metastasis, and local recurrence for colon and rectal cancer

Colon cancer	0 (*n* = 8892)	1 (*n* = 583)	2 (*n* = 271)	3 (*n* = 121)	4 (*n* = 65)	≥5 (*n* = 138)	Total (*n* = 10 070)
**Mortality**							
Alive at follow-up	6454 (72.6)	387 (66.4)	160 (59.0)	76 (62.8)	28 (43.1)	58 (42.0)	7163 (71.1)
Deceased	2410 (27.1)	194 (33.3)	111 (41.0)	45 (37.2)	37 (56.9)	79 (57.2)	2876 (28.6)
Emigrated	28 (0.3)	2 (0.3)	0 (0)	0 (0)	0 (0)	1 (0.7)	31 (0.3)
**Distant metastasis**							
Yes	771 (8.7)	120 (20.6)	76 (28.0)	45 (37.2)	24 (36.9)	63 (45.7)	1099 (10.9)
**Local recurrence**							
Yes	163 (1.8)	24 (4.1)	15 (5.5)	11 (9.1)	3 (4.6)	8 (5.8)	224 (2.2)

Rectal cancer	0 (*n* = 3560)	1 (*n* = 262)	2 (*n* = 109)	3 (*n* = 57)	4 (*n* = 36)	≥5 (*n* = 60)	Total (*n* = 4084)
**Mortality**							
Alive at follow-up	2874 (80.7)	172 (65.6)	63 (57.8)	36 (63.2)	16 (44.4)	28 (46.7)	3189 (78.1)
Deceased	678 (19.0)	90 (34.4)	46 (42.2)	21 (36.8)	20 (55.6)	32 (53.3)	887 (21.7)
Emigrated	8 (0.2)	0 (0)	0 (0)	0 (0)	0 (0)	0 (0)	8 (0.2)
**Distant metastasis**							
Yes	431 (12.1)	76 (29.0)	39 (35.8)	20 (35.1)	17 (47.2)	30 (50.0)	613 (15.0)
**Local recurrence**							
Yes	100 (2.8)	11 (4.2)	11 (10.1)	5 (8.8)	2 (5.6)	8 (13.3)	137 (3.4)

Categorical values are presented as frequency (%).

### Survival analysis

**Fig. 2:**
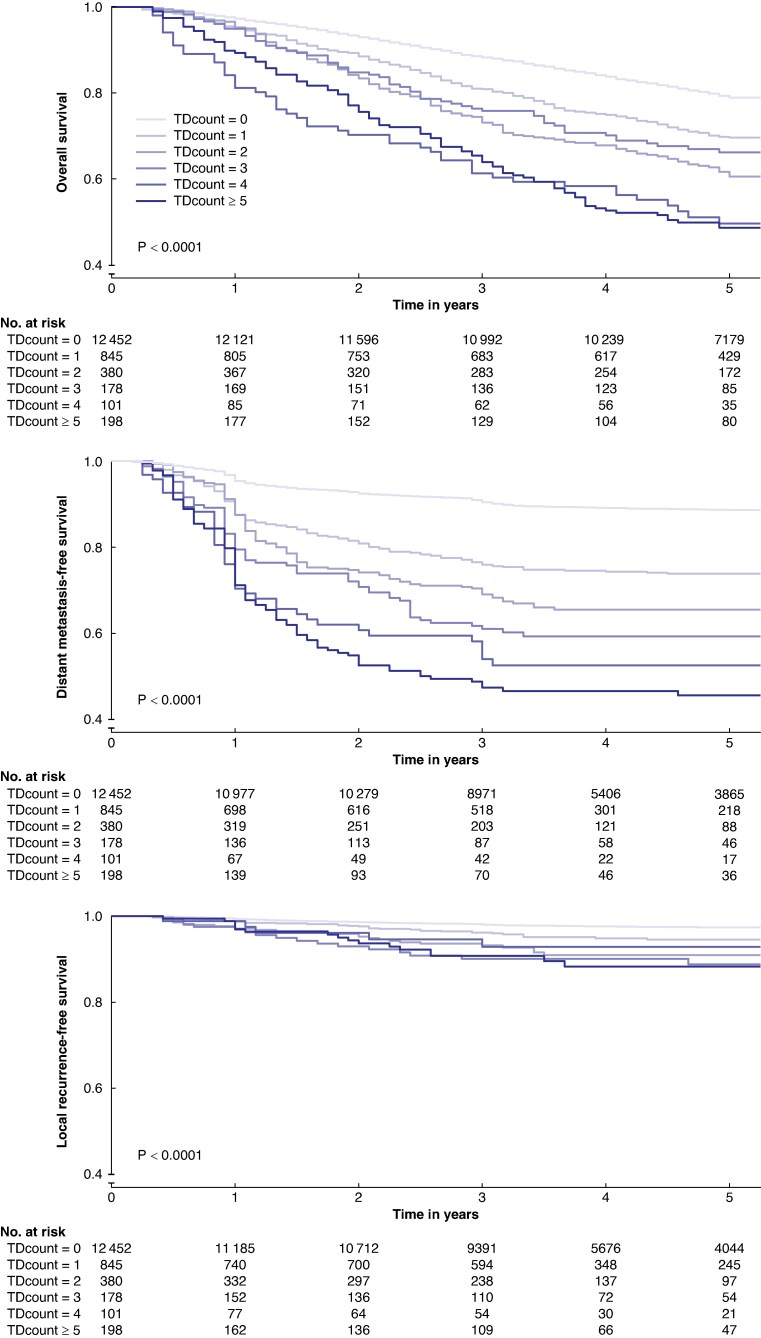
Kaplan–Meier curves of 5-year overall survival, distant metastasis-free survival, and local recurrence-free survival based on tumour deposit count (0, 1, 2, 3, 4 and ≥5) for patients with colorectal cancer Note that the *y*-axis is offset at 40%

*Figure 2* illustrates the association between the tumour deposit count and 5-year overall survival, as well as the 5-year risk of distant metastasis and local recurrence. For 5-year overall survival, the risk of death escalated with an increasing tumour deposit count, ranging from 21% (no tumour deposits) to 51% (≥5 tumour deposits). Similarly, there was a linear stepwise increase in the 5-year risk of distant metastasis, ranging from 11% (no tumour deposits) to 54% (≥5 tumour deposits). For local recurrence the disparity was smaller, ranging from 3% (no tumour deposits) to 12% (≥5 tumour deposits). Subgroup analysis of colon and rectal cancer patients revealed similar incidence in outcomes between groups (*Fig. S2*).

*Table 4* presents uni- and multivariable Cox regression analyses for relative survival, overall survival, distant metastasis, and local recurrence based on tumour deposit count. In the univariable Cox regression analysis, the presence of a single tumour deposit was associated with reduced relative and overall survival, along with increased risk of distant metastasis and local recurrence. Multiple tumour deposits significantly reduced relative and overall survival, and increased distant metastasis and local recurrence compared to both the absence of tumour deposits and a singular tumour deposit. These associations persisted, albeit partially attenuated, in the adjusted Cox regression analysis.

**Table 4:** Hazard ratio of the unadjusted and adjusted effect of tumour deposit count on relative survival, overall survival, distant metastasis and local recurrence

	Relative survival	Overall survival	Distant metastasis	Local recurrence
TD count* (*n* = 14 154)	**EHR**	**HR**	**HR**	**HR**
0	1	1	1	1
1	1.6 (1.5–1.9)	1.5 (1.3–1.7)	2.6 (2.3–3.1)	2.0 (1.4–2.9)
2	2.4 (2.1–2.9)	1.9 (1.7–2.3)	3.6 (3.0–4.3)	3.5 (2.4–5.3)
3	2.5 (1.9–3.2)	1.7 (1.3–2.1)	4.6 (3.5–5.8)	4.7 (2.8–7.7)
4	4.2 (3.2–5.4)	3.2 (2.5–4.2)	6.0 (4.4–8.2)	3.0 (1.3–7.3)
≥5	3.8 (3.1–4.6)	3.0 (2.5–3.6)	7.0 (5.7–8.6)	4.6 (2.8–7.6)
TD count† (*n* *=* 14 040)	**EHR**	**HR**	**HR**	**HR**
0	1	1	1	1
1	1.5 (1.3–1.6)	1.4 (1.3–1.6)	2.4 (2.1–2.8)	1.9 (1.3–2.7)
2	1.9 (1.6–2.3)	1.9 (1.6–2.2)	3.0 (2.5–3.7)	3.1 (2.1–4.6)
3	1.8 (1.4–2.3)	1.8 (1.4–2.3)	3.6 (2.8–4.6)	3.8 (2.3–6.4)
4	3.1 (2.4–4.9)	3.1 (2.3–4.0)	4.6 (3.4–6.3)	2.4 (1.0–5.8)
≥5	2.5 (2.1–3.0)	2.4 (2.0–2.9)	4.4 (3.6–5.5)	3.3 (2.0–5.5)

TD = tumour deposit. EHR = excess hazard ratio. All data are presented as (excessive) HR (95% c.i.).

^*^Unadjusted.

^†^Adjusted for age, sex, number of positive lymph nodes and neoadjuvant treatment.

Multivariable Cox regression sensitivity analysis of the effect of tumour deposit count is presented in *Table S3*. All regressions, except for relative survival, were based on pooled data from the imputation analysis. In the sensitivity analysis, tumour deposit count remained significantly associated with worse hazard ratios for relative survival, overall survival, and distant metastasis, but not for local recurrence.

## Discussion

The present study is the first study to show a strong negative association between tumour deposit count and long-term oncological outcomes, independent of lymph node status. These findings advocate for considering both the presence of tumour deposits in lymph node-positive CRC, and the occurrence of multiple tumour deposits in CRC staging protocols.

This study contained a large sample of patients, of which 12% had tumour deposits. This is slightly less than the reported prevalence of 20%, which may be reflective of the stricter definition implemented in TNM8^6^. Nearly all patients were discussed at a preoperative multidisciplinary conference. Only 29% of patients with tumour deposits were staged as N1c, emphasizing the effect of omitting tumour deposits in lymph node-positive patients.

A stepwise negative association between tumour deposit count and relative survival, overall survival, distant metastasis, and local recurrence in univariable and adjusted models was observed. Tumour deposit counts were divided into groups (0, 1, 2, 3, 4, and ≥5) to intuitively depict how a stepwise increase in tumour deposit count affects the risk of adverse outcomes. Although the relationship between tumour deposit count and the risk of local recurrence was not as prominent as for the other outcomes, these findings indicate that multiple tumour deposits are associated with worse prognosis for all investigated oncological outcomes. Additionally, the presence of tumour deposits emerged as a negative prognostic factor even in the presence of positive lymph nodes.

The importance of tumour deposits has been demonstrated both in colon and rectal cancer cohorts^8,9^. In this study, we found that tumour deposit count is prognostic even after adjusting for age, sex, neoadjuvant treatment, and number of positive lymph nodes. When accounting for other histopathological high-risk factors in the sensitivity analysis, the hazard ratio for all outcomes was notably reduced but remained significant for relative survival, overall survival, and distant metastasis, but not for local reccurence. This suggests that tumour deposits may not directly influence the risk of local recurrence but are instead indirectly linked with local recurrence by their association with other adverse histopathological risk factors, which is in line with earlier studies and the proposed metastatic pathway for tumour deposits^8,10,24,25^. However, this observation may also be attributed to the limited number of local recurrences, which restricts statistical strength. Notably, 80% of all recurrences occur within 3 years of curative surgery, indicating that the follow-up period should not have been a limiting factor^26^. Instead, the low number of local recurrences might be reflective of the effectiveness of current treatments in reducing the risk of local recurrence, supported by the consistently low rates across all investigated groups. This highlights the need to focus on pathways for distant metastasis to improve outcomes. Tumour deposit count was also shown to have equivalent associated effects on colon and rectal cancer patients. CRC was previously seen as a homogenous group, but recent research has challenged this view and called for separate analysis^27^. This study shows that not only presence of tumour deposits but the number is important in both colon and rectal cancer cohorts, expanding on results from earlier studies^8,9^.

The Swedish Colorectal Cancer Registry is a comprehensive database but faces challenges with missing data. Some gaps arise from updates in the variable list, with older, broader variables being replaced by more precise subclassifications. In the current data set, subclassification of lymphatic/vascular infiltration, such as EMVI, was missing for patients operated on in 2016. To address this, imputation was performed in consultation with a professional statistician, ensuring rigorous statistical integrity. Other missing unregistered data, such as smoking status, family history, and red meat consumption, can introduce unobserved confounders. User errors, like misreporting weight and height, also contributed to missing data. In this study clear errors were corrected and unclear errors excluded. Lastly, as histopathological data from the registry were retrieved from all hospitals in Sweden, re-review by a dedicated pathologist was not possible.

This study spans four years (2016–2019), during which time there was a transition from the seventh to the eighth edition of the TNM staging system, as well as changes in CRC treatment protocols. To mitigate potential bias stemming from more effective staging or treatments, the year of surgery was included in the sensitivity analysis. Finally, as this study included patients up until 2019, 5-year follow-up was not feasible for all patients at the time of data extraction, resulting in a significant number of censored patients in the fourth and fifth year in the Kaplan–Meier curves, potentially limiting long-term insights.

The current TNM staging system does not incorporate valuable prognostic information related to tumour deposits. This gap is consistent with prior research and underscores a growing call for a revised staging system that integrates these data to enhance prognostic accuracy and better guide adjuvant treatment decisions^6–8,15,28–30^. Efforts to stratify tumour deposit counts into distinct prognostic categories have shown potential to refine CRC staging, but a consensus on optimal stratification has yet to be reached^31–37^. Discrepancies between studies may stem from limitations in data sets, variations in outcomes studied, or inconsistencies in diagnostics.

The present study utilizes histologically diagnosed tumour deposits as a prognostic factor, and therefore any impact on oncological outcomes may have already occurred by the time of surgery. Recent studies have explored the potential of preoperative radiological diagnosis of tumour deposits with promising results^38–40^. Early detection of tumour deposits through imaging could deepen our understanding of their role in metastasis, potentially enabling personalized treatment.

Although preoperative detection of tumour deposits holds promise for improving prognostic assessment, it may not yet translate into better patient outcomes within the framework of currently available oncological treatments. The high incidence of adverse outcomes observed in this study suggests that existing treatments are insufficient to mitigate the risks associated with tumour deposits. Effective treatment strategies remain challenging, partly due to the unknown aetiology of tumour deposits which limits both diagnostic and therapeutic options^41^. Genetic and epigenetic research could help unravel the origins of tumour deposits and identify novel oncological targets, potentially improving outcomes for this patient group.

This study demonstrated that tumour deposit count is a negative prognostic factor for overall survival and distant metastasis in CRC, independent of the number of positive lymph nodes. These findings challenge the omission of tumour deposits in lymph node-positive CRC in the current TNM system, and advocate for integration into future staging systems.

## Supplementary Material

znae309_Supplementary_Data

## Data Availability

All data used are available through the Swedish Colorectal Cancer Registry.

## Previous communication

Abstract presented at the Swedish Surgical Week 2024 in Karlstad, Sweden (19–23 August 2024) and at the European Society of Coloproctology’s 19th Scientific and Annual Conference in Thessaloniki, Greece (25–27 September 2024).
