# Correction to: Tumour deposit count is an independent prognostic factor in colorectal cancer—a population-based cohort study

**DOI:** 10.1093/bjs/znaf135

**Published:** 2025-06-24

This is a correction to: Simon Lundström, Erik Agger, Marie-Louise Lydrup, Fredrik Jörgren, Pamela Buchwald, Tumour deposit count is an independent prognostic factor in colorectal cancer—a population-based cohort study, *British Journal of Surgery*, Volume 112, Issue 1, January 2025, znae309, https://doi.org/10.1093/bjs/znae309

In the original publication of the article, there was a discrepancy between the result text and Table 1 due to unclear phrasing. Although both TD-positive and TD-negative tumors were more commonly located in the left colon than the right, right-sided tumors were relatively more frequent among TD-positive patients. As tumor location was included as a variable in the regression analysis, this ambiguity did not affect the results.

In section **Results**, **Study Population**, text for *Table 1* should read: “While both TD-positive and TD-negative tumors were more commonly left-sided, the proportion of right-sided tumors was higher among TD-positive cases compared to TD-negative cases.” instead of: “Tumour deposit-positive colon cancers were more frequently located in the right colon.”

The emendation was made in the article.

