# Supplementary material for: Tumour deposit count is an independent prognostic factor in colorectal cancer—a population-based cohort study
Source: Br J Surg. 2024 Dec 30;112(1):znae309. doi: 10.1093/bjs/znae309 (PMC11683728; doi:10.1093/bjs/znae309)
Supplement: znae309_Supplementary_Data [file znae309_supplementary_data.zip › Supplementary_Material.docx]

Tumour deposit count is an independent prognostic factor in colorectal cancer – a population-based cohort study

Authors: Simon Lundström^1,2^, Erik Agger^1, 2^, Marie-Louise Lydrup^1, 2^, Fredrik Jörgren^2, 3^, Pamela Buchwald^1, 2^

^1^ Department of Surgery, Skåne University Hospital, Malmö, Sweden

^2^ Department of Clinical Sciences, Lund University, Lund, Sweden

^3^ Department of Surgery, Helsingborg Hospital, Lund University, Helsingborg, Sweden

**Corresponding author**: Simon Lundström^1,2^ (Lundström, S), MD, PhD-student.
Email: Simon.Lundstrom@med.lu.se, **ORCID ID**: 0000-0002-5602-7128,
Address: Department of Surgery, Skåne University Hospital, SE-214 21, Malmö
Telephone: +4670-6686093. Twitter @Simon_Lundstrom

**Supplementary Materials - Index**

| **Supplementary Methods** |  |
| --- | --- |
| Definitions | *page 2* |
| **Supplementary Figures and Tables** |  |
| Supplementary Figure S1. Direct Acyclic Graph | *page 3* |
| Supplementary Figure S2. Subgroup survival analysis | *page 4* |
| Supplementary Table S3. Sensitivity analysis | *page 5* |
| **References** | *page 6-7* |
|  |  |

**Supplementary Methods**

**Definitions**

*Abdominal surgery* was defined as total colectomy, left-, right-, transverse-, and sigmoid hemicolectomy, anterior resection, rectal resection, Hartman’s resection, and abdominoperineal resection. Patients were included independent of open or minimally invasive surgical approach. Appendectomy, ileocecal resection, local excision, and laparotomy without resection were excluded.

*Colon cancer* was defined as adenocarcinoma located from the proximal ascending colon to the distal segment of sigmoid. Tumor location was classified as right-sided if located proximal to the splenic flexure and left-sided if located at or distal to the splenic flexure.

*Rectal cancer* was defined as adenocarcinoma located 15 cm from the anal verge measured by rigid sigmoidoscopy.

*Non-radical resection* was defined as R2-surgery, 0.0 mm circumferential resection margin, or 0.0 mm distal resection margin. *Tumour grade* was divided into low grade (well to moderately differentiated) and high grade (poorly differentiated). *Lymphovascular invasion* was defined as tumour infiltration of lymphatic or venous vessels, irrespective of size and intra- or extramural location.

**Supplementary Figures and Tables**

Supplementary Figure S1

**Direct Acyclic Graphs** plot for overall survival, distant metastasis, and local recurrence. Data points are derived from literature and expert knowledge. Nodes represent variables and arrows represent casual associations. Green-coloured nodes represent exposure. Red-coloured nodes represent ancestors of exposure. Blue-coloured nodes represent ancestors of outcomes. Red lines represent biasing paths whereas green lines represent causal paths. OS = overall survival. DM = distant metastasis. LR = local recurrence.

Supplement Figure S2

Kaplan-Meier curves of 5-years local recurrence-free survival (top row), distant metastasis-free survival (middle row), and overall survival (bottom row) based on TD-count (0, 1, 2, 3, 4 and ≥5) for patients with colon (left) and rectal (right) cancer. Note that the y-axis is offsetted at 40%.

Supplementary Table S3

Hazard ratio of the sensitivity analysis of the effect of tumour deposit count on on relative survival, overall survival, distant metastasis, and local recurrence.

| \|  \| **Relative Survival** \| **Overall Survival** \| **Distant metastasis** \| \| **Local recurrence** \| \| \| --- \| --- \| --- \| --- \| --- \| --- \| --- \| \| TD-count \| EHR \| HR \| HR \| HR \| \| \| \| 0 \| 1 \| 1 \| 1 \| 1 \| \| \| \| 1 \| 1.2 (1.0-1.3) \| 1.1 (1.0-1.3) \| 1.2 (1.0-1.4) \| 1.0 (0.7-1.4) \| \| \| \| 2 \| 1.5 (1.2-1.8) \| 1.4 (1.2-1.7) \| 1.4 (1.2-1.8) \| 1.5 (0.9-2.2) \| \| \| \| 3 \| 1.6 (1.2-2.1) \| 1.4 (1.1-1.8) \| 1.7 (1.3-2.2) \| 1.9 (1.1-3.2) \| \| \| \| 4 \| 2.5 (1.9-3.4) \| 2.3 (1.7-3.0) \| 2.1 (1.5-2.9) \| 1.1 (0.4-2.6) \| \| \| \| ≥ 5 \| 1.9 (1.5-2.4) \| 1.8 (1.5-2.2) \| 2.1 (1.6-2.6) \| 1.4 (0.8-2.4) \| \| \| \|  \|  \|  \|  \| \|  \| |
| --- | --- | --- | --- | --- | --- | --- | --- | --- | --- | --- | --- | --- | --- | --- | --- | --- | --- | --- | --- | --- | --- | --- | --- | --- | --- | --- | --- | --- | --- | --- | --- | --- | --- | --- | --- | --- | --- | --- | --- | --- | --- | --- | --- | --- | --- | --- | --- | --- | --- | --- | --- | --- | --- | --- | --- | --- | --- | --- | --- | --- | --- | --- |

*TD = tumour deposit. EHR = excess hazard ratio. HR = hazard ratio. All data is presented as Hazard Ratio (95% confidence interval). Sensitivity multivariable cox regression of tumour deposit-counts effect on relative survival, overall survival, distant metastasis and local recurrence. Adjusted for age, sex, clinical stage, neoadjuvant treatment, pathological stage, tumour grade, numbers of positive lymph nodes, perineural growth, vascular/lymphatic invasion, EMVI and the year of diagnosis to adjust for differences in TNM-staging. For relative survival, EHR could not be combined with imputation and EMVI was therefore not included in adjustments.*

**References**

1. Eileen M, Melina A, Gini A, Lorenzoni V, Cabasag CJ, Mathieu L, et al. Global burden of colorectal cancer in 2020 and 2040: incidence and mortality estimates from GLOBOCAN. Gut. 2023;72(2):338.

2. Brierley J GM, Wittekind C. TNM Classification of Malignant Tumours (8th edn). Chichester: Wiley-Blackwell; 2017.

3. Jiang Y, Yuan H, Li Z, Ji X, Shen Q, Tuo J, et al. Global pattern and trends of colorectal cancer survival: a systematic review of population-based registration data. Cancer Biol Med. 2021;19(2):175-186.

4. Hemminki K, Försti A, Hemminki A. Survival in colon and rectal cancers in Finland and Sweden through 50 years. BMJ Open Gastroenterol. 2021;8(1).

5. Sobin LH, Gospodarowicz MK, C W. TNM Classification of Malignant Tumours (7th edn). Chichester: Wiley-Blackwell; 2009.

6. Ueno H, Nagtegaal ID, Quirke P, Sugihara K, Ajioka Y. Tumor deposits in colorectal cancer: Refining their definition in the TNM system. Ann Gastroenterol Surg. 2023;7(2):225-235.

7. Nagtegaal ID, Knijn N, Hugen N, Marshall HC, Sugihara K, Tot T, et al. Tumor Deposits in Colorectal Cancer: Improving the Value of Modern Staging—A Systematic Review and Meta-Analysis. Journal of Clinical Oncology. 2017;35(10):1119-1127.

8. Jörgren F, Agger E, Lydrup M-L, Buchwald P. Tumour deposits in colon cancer predict recurrence and reduced survival in a nationwide population-based study. BJS Open. 2023;7(6).

9. Agger E, Jörgren F, Jöud A, Lydrup M-L, Buchwald P. Negative Prognostic Impact of Tumor Deposits in Rectal Cancer – A National Study Cohort. Annals of Surgery. 2022:10.1097/SLA.0000000000005755.

10. Lundström S, Agger E, Lydrup ML, Jörgren F, Buchwald P. Adverse impact of tumor deposits in lymph node negative rectal cancer - a national cohort study. Int J Colorectal Dis. 2023;38(1):66.

11. Doroudian S, Osterman E, Glimelius B. Risk Factors for Recurrence After Surgery for Rectal Cancer in a Modern, Nationwide Population-Based Cohort. Annals of Surgical Oncology. 2024.

12. Nagtegaal ID, Quirke P. Colorectal tumour deposits in the mesorectum and pericolon; a critical review. Histopathology. 2007;51(2):141-149.

13. Betge J, Pollheimer MJ, Lindtner RA, Kornprat P, Schlemmer A, Rehak P, et al. Intramural and extramural vascular invasion in colorectal cancer. Cancer. 2012;118(3):628-638.

14. Lino-Silva LS, Xinaxtle DL, Salcedo-Hernández RA. Tumor deposits in colorectal cancer: the need for a new “pN” category. Annals of Translational Medicine. 2020;8(12):733.

15. Wu W, Zeng S, Zhang X, Liu P, Qiu T, Li S, et al. The value of tumor deposits in evaluating colorectal cancer survival and metastasis: a population-based retrospective cohort study. World Journal of Surgical Oncology. 2022;20(1):41.

16. Moberger P, Sköldberg F, Birgisson H. Evaluation of the Swedish Colorectal Cancer Registry: an overview of completeness, timeliness, comparability and validity. Acta Oncologica. 2018;57(12):1611-1621.

17. Osterman E, Hammarström K, Imam I, Osterlund E, Sjöblom T, Glimelius B. Completeness and accuracy of the registration of recurrences in the Swedish Colorectal Cancer Registry (SCRCR) and an update of recurrence risk in colon cancer. Acta Oncologica. 2021;60(7):842-849.

18. Lord AC, Knijn N, Brown G, Nagtegaal ID. Pathways of spread in rectal cancer: a reappraisal of the true routes to distant metastatic disease. Eur J Cancer. 2020;128:1-6.

19. Brouwer NPM, Oguz Erdogan AS, van Vliet S, Rutgers N, Knijn N, van Lijnschoten G, et al. Unraveling the routes to distant metastases in colorectal cancer: Tumor deposits and lymph node metastases as the gateway. Cancer Communications. 2024;44(10):1209-1213.

20. Ryu HS, Kim J, Park YR, Cho EH, Choo JM, Kim JS, et al. Recurrence Patterns and Risk Factors after Curative Resection for Colorectal Cancer: Insights for Postoperative Surveillance Strategies. Cancers (Basel). 2023;15(24).

21. Tamas K, Walenkamp AM, de Vries EG, van Vugt MA, Beets-Tan RG, van Etten B, et al. Rectal and colon cancer: Not just a different anatomic site. Cancer Treat Rev. 2015;41(8):671-679.

22. Long Q, Xu Y, Ma G, Mao W. Prognostic Value of Tumor Deposit Counts in Patients with Stage III Colorectal Cancer: A Population-Based Study. Journal of Investigative Surgery. 2022;35(7):1502-1509.

23. Delattre J-F, Selcen Oguz Erdogan A, Cohen R, Shi Q, Emile J-F, Taieb J, et al. A comprehensive overview of tumour deposits in colorectal cancer: Towards a next TNM classification. Cancer Treatment Reviews. 2022;103:102325.

24. Moon JY, Lee MR, Ha GW. Prognostic value of tumor deposits for long-term oncologic outcomes in patients with stage III colorectal cancer: a systematic review and meta-analysis. International Journal of Colorectal Disease. 2022;37(1):141-151.

25. Pricolo VE, Steingrimsson J, McDuffie TJ, McHale JM, McMillen B, Shparber M. Tumor Deposits in Stage III Colon Cancer: Correlation With Other Histopathologic Variables, Prognostic Value, and Risk Stratification-Time to Consider "N2c". Am J Clin Oncol. 2020;43(2):133-138.

26. Wu WX, Zhang DK, Chen SX, Hou ZY, Sun BL, Yao L, et al. Prognostic impact of tumor deposits on overall survival in colorectal cancer: Based on Surveillance, Epidemiology, and End Results database. World J Gastrointest Oncol. 2022;14(9):1699-1710.

27. Wang S, Guan X, Ma M, Zhuang M, Ma T, Liu Z, et al. Reconsidering the prognostic significance of tumour deposit count in the TNM staging system for colorectal cancer. Scientific Reports. 2020;10(1):89.

28. Pei JP, Zhang CD, Fu X, Ba Y, Yue S, Zhao ZM, et al. A Modified Tumor-Node-Metastasis Classification for Stage III Colorectal Cancers Based on Treating Tumor Deposits as Positive Lymph Nodes. Front Med (Lausanne). 2020;7:571154.

29. Karbanowicz E, Fuchs TL, Chou A, Sioson L, Sheen A, Ahadi MS, et al. What are the problems with the current staging of discontinuous tumour nodules (DTNs) in colorectal carcinoma? Is there a better way? Pathology. 2022;54(7):848-854.

30. Pei J-P, Zhang C-D, Liang Y, Zhang C, Wu K-Z, Li Y-Z, et al. A Modified Pathological N Stage Including Status of Tumor Deposits in Colorectal Cancer With Nodal Metastasis. Frontiers in Oncology. 2020;10.

31. Liu F, Zhao J, Li C, Wu Y, Song W, Guo T, et al. The unique prognostic characteristics of tumor deposits in colorectal cancer patients. Ann Transl Med. 2019;7(23):769.

32. D'Souza N, Shaw A, Lord A, Balyasnikova S, Abulafi M, Tekkis P, et al. Assessment of a Staging System for Sigmoid Colon Cancer Based on Tumor Deposits and Extramural Venous Invasion on Computed Tomography. JAMA Netw Open. 2019;2(12):e1916987.

33. Lord AC, Moran B, Abulafi M, Rasheed S, Nagtegaal ID, Terlizzo M, et al. Can extranodal tumour deposits be diagnosed on MRI? Protocol for a multicentre clinical trial (the COMET trial). BMJ Open. 2020;10(10):e033395.

34. Lord AC, D’Souza N, Shaw A, Rokan Z, Moran B, Abulafi M, et al. MRI-Diagnosed Tumor Deposits and EMVI Status Have Superior Prognostic Accuracy to Current Clinical TNM Staging in Rectal Cancer. Annals of Surgery. 2022;276(2).

35. Lord A, Brown G, Abulafi M, Bateman A, Frankel W, Goldin R, et al. Histopathological diagnosis of tumour deposits in colorectal cancer: a Delphi consensus study. Histopathology. 2021;79(2):168-175.
